# The three stages of polytrauma rehabilitation– a recommendation and a systematic literature review on behalf of SICOT

**DOI:** 10.1007/s00264-024-06385-0

**Published:** 2024-12-16

**Authors:** Felix Karl-Ludwig Klingebiel, Vincent Landre, Morgan Hasegawa, Yannik Kalbas, Marc Hanschen, Kenichi Sawauchi, Sayid Omar Mohamed, Mohammed Zarti, Mohammad Zain-ur-Rehmann, Alaric Aroojis, Shanmuganathan Rajasekaran, Hans-Christoph Pape, Roman Pfeifer, on behalf of SICOT Trauma & Rehabilitation Research Group, Sakti Prasad Das, Patrick Herard, Gleb Korobushkin, Caterina Pasquale, Denis Yuen

**Affiliations:** 1 University of Zurich, Zurich, Switzerland; 2 Harald-Tscherne Laboratory for Orthopaedic and Trauma Research, University Hospital Zurich, Switzerland; 3 University of Hawai‘i, Honolulu, USA; 4 Technical University of Munich, Klinikum rechts der Isar, Munich, Germany; 5 Kobe University Graduate School of Medicine, Kobe, Japan; 6 Jazeera University Hospital, Mogadishu, Somalia; 7 Alkhadra Hospital, Tripoli, Libya; 8 Worcestershire Acute Hospitals NHS Trust, Worcester, UK; 9 Bai Jerbai Wadia Hospital for Children, Mumbai, India; 10 Ganga Medical Centre and Hospitals Pvt. Ltd, Coimbatore, India

**Keywords:** Polytrauma, Rehabilition, Recovery

## Abstract

**Purpose:**

Polytrauma presents a devastating event with great impact on the patient’s life. While we are taking great care of improving our treatment algorithms, the rehabilitation often takes place outside of our direct field of vision. Yet, adequate rehabilitation is crucial for the patients to regain their former lives. The aim of this study, on the behalf of SICOT Trauma & Rehabilitation Research Group, was to identify rehabilitation strategies and standards in existing scientific literature.

**Methods:**

A systematic literature search of MEDLINE and Embase from 2000 to 2023 was conducted. Inclusion criteria was the description of polytrauma rehabilitation strategies in the acute, post-acute or long-term stage. Reported treatment aims, conducted therapies and challenges were extracted and stratified to either of the stages.

**Results:**

A total of 5212 studies were identified and 6 reviews and one original study were included according to our criteria. Overall, no article of higher evidence on how to perform polytrauma rehabilitation could be identified. From the available literature, disciplines involved in the rehabilitation could be described such as major challenges along the rehabilitation process.

**Conclusion:**

This study highlights the need for standardized polytrauma rehabilitation algorithms. Whereas we could identify important information about each rehabilitation stage, we did not encounter specific evidence for prioritization of different therapies or algorithms of treatment. Polytrauma rehabilitation needs to shift from eminence to evidence.

**Supplementary Information:**

The online version contains supplementary material available at 10.1007/s00264-024-06385-0.

## Introduction

Polytrauma presents a devastating event for the patient and their family and greatly affects multiple aspects of their lives. Even after the clinical treatment has finished, the aftermath of this severe trauma is still noticeable in a reduction of quality of life and a long road ahead to recover and reintegrate into everyday life. In addition, besides the direct socioeconomic burden that is caused by the cost of the extensive medical treatment, indirect expenses that result from the inability to work, encumber the health system.

To regain the best possible long-term function with re-entry into work and re-integration into social life as well as to prevent pulmonary and cardiovascular complications, an immediate beginning of so-called acute-rehabilitation - adapted to the patients’ injury pattern and physiological function - is required. This should be stepwise readjusted to the patients’ potential as surgical reconstruction is proceeded and they regain musculoskeletal capability. Next to musculoskeletal rehabilitation, especially in the early stage, the patient might benefit from further adjunctive rehabilitation such as pulmonary and psychological therapy. After the surgical reconstruction and medical treatment is finished, the patient should be transferred to a specialized rehabilitation facility to perform post-acute rehabilitation respective the persisting limitations for the musculoskeletal system i.e. weight bearing and movement limits (post-acute rehabilitation). After completed post-acute rehabilitation, the patient is discharged to their home or – depending on the age and injury pattern – to a care facility where late-rehabilitation is advised to guide the patients’ long-term recovery.

Even though this part of treatment is most essential for the patient and the long-term outcome, there seems to be not much evidence that compares different rehabilitation strategies in the literature [1, 2]. This results in a non-standardized rehabilitation process and most institutions have developed their own algorithm that depends on their human and overall resources [3]. Topics of utmost importance hereby would be the composition of the three different stages of rehabilitation as which disciplines are required and which treatment aims consist for each of those. In some hospitals, acute-rehabilitation is difficult to properly perform since it requires multidisciplinary team of rehabilitation/physiotherapy specialists that consult the patients on a frequent basis. Another pitfall seems to be the persistence of late-rehabilitation as the patient is back home and multiple factors play a role for successful integration of rehabilitation in their everyday life.

The impetus of our study was to collect information from the existing literature about the composition of different stages of polytrauma rehabilitation and to generate an interdisciplinary recommendation for the rehabilitation process.

## Materials & methods

The reporting of this systematic review follows the Preferred Reporting Items for Systematic Reviews and Meta-Analyses (PRISMA) guidelines (http://www.prisma-statement.org/).

### Eligibility criteria

Inclusion criteria were original studies or reviews published between 2000 and 2023 that focus on at least one of the three stages of rehabilitation for polytraumatized patients and provide detailed descriptions for clinical practice. Exclusion criteria were paediatric trauma, combat injuries, isolated injuries, and insufficient description of the rehabilitation carried out.

### Information sources and search strategy

A systematic search of MEDLINE and Embase was conducted on 19 April 2023. A combination of controlled vocabulary and regular search terms were used in combination with MESH/EMTREE terms. Additional sources either were identified outside the systematic search strategy as literature from other publications or were recommended by experts in the field. An overview of the search terms can be found in the supplementary information (SI).

### Selection process

All identified publications were independently screened by FKLK, VL, MH and SOM in a blinded fashion. Discrepancies were resolved in regular consensus meetings. Articles were screened, analyzed and stored as PDF files in EndNote™ version 20 by Carivate™.

### Data item

The three stages of rehabilitation are frequently referred to in literature as (1) Acute rehabilitation, (2) Post-acute rehabilitation and (3) Late rehabilitation. Acute rehabilitation hereby describes the initial in-hospital rehabilitation treatment. Post-acute rehabilitation refers to rehabilitation in a specialized facility that the patient is transferred to after clinical/surgical treatment is completed. Late rehabilitation describes the rehabilitation that the patient receives after discharge from the rehabilitation facility to their home and is conducted in an outpatient institution.

Parameters of interest were aims, therapies/involved disciplines and challenges described in relation to the regarding rehabilitation stage.

### Synthesis methods

Data was collected manually by the first authors and transferred onto an Excel spreadsheet. Besides general information and year of publication, the focus was on the treatment aims, conducted therapies/exercises, involved disciplines and challenges from each individual rehabilitation stage. Relevant information to these topics were stratified to the respective rehabilitation stage. In an interdisciplinary approach, members of the SICOT Trauma Research Group and the Rehabilitation section of SICOT analyzed the collected information and created a rehabilitation algorithm for the polytraumatized patients.

## Results

**Fig. 1:**
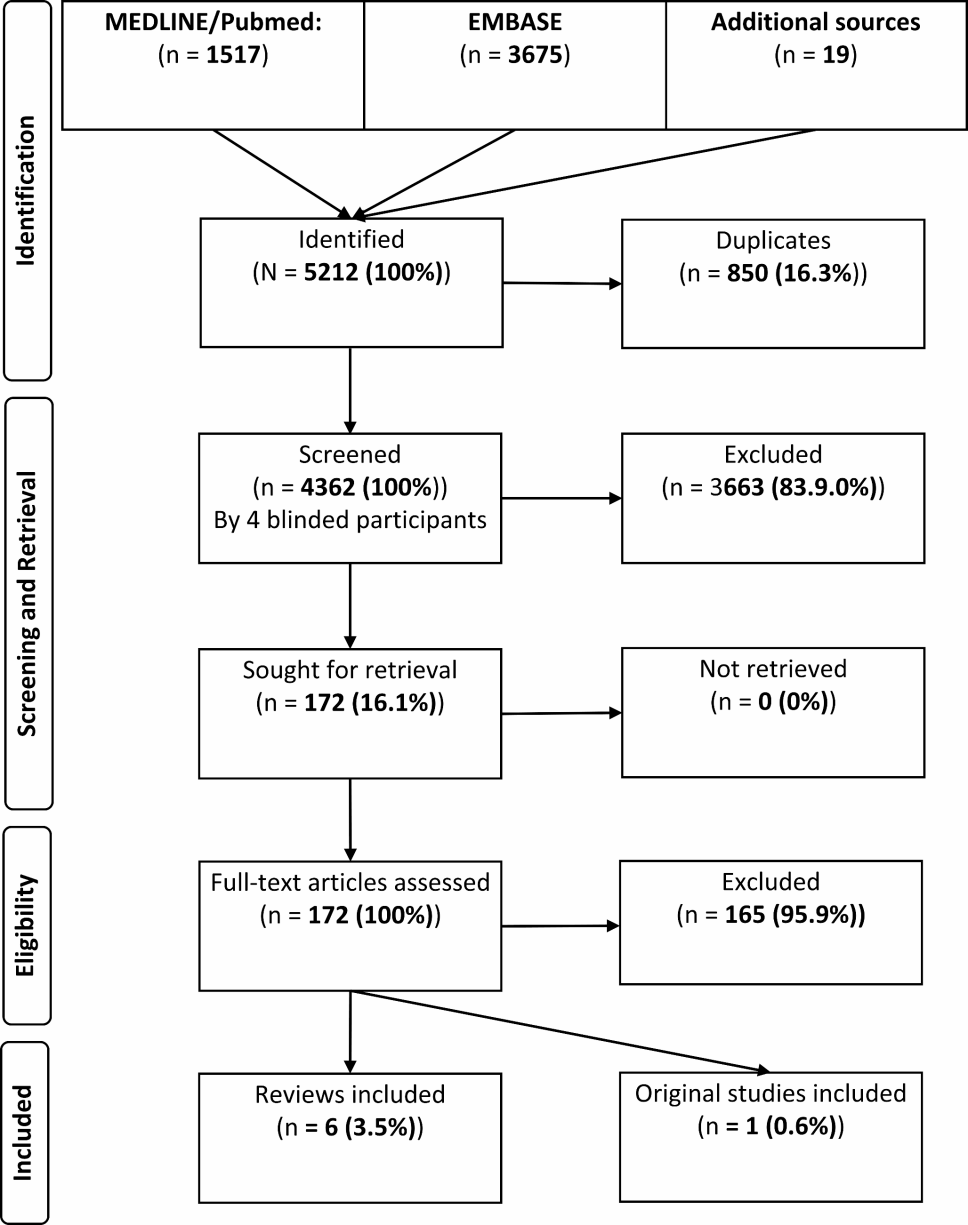
Flowchart systematic Literature search

A total of 5212 publications were identified, of which 850 articles were excluded as duplicates. 4362 articles were screened, of which 172 were retrieved and were assessed for full article screening. One original study (survey) and six reviews met our inclusion criteria (Table 1; Fig. 1). Four articles focused on the acute phase, one on the post-acute phase and one on the late phase whereas one publication refereed to either of those stages.

**Table 1:** Overview of included articles

Author	Year	Journal	Publication type
Simmel [4]	2010	Trauma und Berufskrankheit	Review
Simmel [5]	2011	Orthopädische Praxis	Review
Simmel et al. [6]	2013	Der Chirurg	Review
Debus et al. [7]	2014	Rehabilitation	Survey
Von Matthey et al. [8]	2015	Der Orthopäde	Review
Jang et al. [9]	2019	Acute and Critical Care	Review
Critchfield et al. [10]	2019	Physical Medicine and Rehabilitation Clinics of North America	Review

There were no retrospective or prospective clinical studies that provide evidence on the conduction of polytrauma rehabilitation. The highest evidence on this topic provided was a survey on in-house rehabilitation protocols by Debus et al. [7]. Yet no clinical studies seem to have been performed on this topic. This finding underlines that there is a clear lack of evidence in the literature on polytrauma rehabilitation. There is need for standardized studies evaluating treatment approaches and protocols to optimize that patients’ outcome after severe trauma.

### Aims

In the acute rehabilitation, one the main aims of the treatment is the recovery of physical function and improvement of mobility next to preventing secondary complications (i.e. thromboembolic events / pulmonary complications) that are associated with long-term mobilization. Restoration of the quality of life already seems to be an important factor that is addressed early in the acute rehabilitation, with the focus on the patient`s return back to normal life with social as well as vocational reintegration. In an individual/tailored approach, the patient`s rehabilitation potential is improved that he benefits best from the upcoming journey to recovery. Psychological support is also used to cope with the traumatic experiences. In this phase, the diligent coordination of the entire rehabilitation process to ensure seamless transfer to the next rehabilitation phase.

In the post-acute rehabilitation there seems to be special attention towards to a holistic social and vocational rehabilitation, accompanied by psychological support.

The late phase presents a combination from functional rehabilitation, psychological support, social and vocational rehabilitation, paired with the prevention of late complications. Depending on the patient`s demands, pain and other managements can be performed (Table 2). Yet it seems like the aims need to be always adapted to the patient’s injury pattern and individual goals.

**Table 2:** Treatment aims during the rehabilitation stages

	Aspect	Acute stage					Post-acute stage		Long-term stage	
	Authors/Publications	Debus et al.	Jang et al.	Matthey et al.	Simmel et al. 2010	Simmel et al. 2013	Critchfield et al.	Matthey et al.	Matthey et al.	Simmel et al. 2011
Physical Function/ Mobility	Regaining physical mobility/function	X		X	X	X			X	X
	Communication/swallowing skills									X
QoL	Long term improvement quality of life		X						X	
Psychological State	Psychological care (Patient)				X		X		X	
	Psychological care (Relatives)					X				
Social/ Vocational reintegration	Vocational rehabilitation/reintegration				X		X	X	X	
	Social reintegration				X			X	X	X
Return to normal life	Return to normal life/autonomy		X							X
	Management of complex life skills						X			
Prevention Conplications /Dependencies	Prevent chronic conditions (disabilities/dependencies)				X	X				
	Prevent secondary consequences/complications			X						X
	Improving patients prognosis		X							
Rehabilitation potential	Achieving rehabilitation capability (mobility and independence)	X				X				
Invidualized care	Individualized Care				X	X				
Ressources and coordination	Coordination physical/psychological treatment/resources	X				X				
	Implementation curative/preventive measures and predictors			X		X				
	Organizing seamless rehabilitation phases and early transfer					X				
Other	Pain management									X

### Therapies/Involved disciplines

Rehabilitation is a multidisciplinary task that encompasses multiple fields of expertise and professionals.

In the acute rehabilitation, especially occupational and physiotherapy next to speech therapy and psychological support seem to be required. In addition, disciplines as art/music therapy, social work and the rehabilitation management are mentioned in the literature. Next to more specific disciplines such as dysphagia/respiratory therapy and vocational rehabilitations.

Similar aspects are mentioned in the post-acute phase, whereas the reintegration into society and work with social workers etc. gets more focused compared to the prior phase.

In the long-term rehabilitation, psychological work-up and social work seems to be of specific relevance next to the individual physical rehabilitation. A complete overview of the different involved disciplines is provided in Table 3.

**Table 3:** Therapies / involved disciplines during the rehabilitation stages

Therapy	Acute stage					Post-acute stage		Long-term stage	
Authors/Publications	Debus et al.	Jang et al.	Matthey et al.	Simmel et al. 2010	Simmel et al. 2013	Critchfield et al.	Matthey et al.	Matthey et al.	Simmel et al. 2011
Physiotherapy	X	X	X	X	X	X	X	X	
Occupational Therapy	X	X	X	X	X	X	X		X
Dysphagia therapy	X								
Speech therapy	X			X		X			X
Vocational rehabilitation					X		X	X	
Respiratory therapy		X							
Hydrotherapy	X								
Kinesiotherapy						X			
Art/Music therapy	X			X					
Nursing therapy				X		X			
Psychology			X	X	X	X		X	X
Rehabilitation physicians					X	X	X		
Trauma surgeons					X				X
Clinical pharmacist						X			
Recreational therapy						X			
Vision therapy						X			
Chaplain						X			
Dietician						X			
Quality Management									X
Public health									X
Rehabilitation management			X		X			X	
Social work			X		X	X	X	X	X
Social insurance providers					X				

### Aspects with improvement potential/Pitfalls in the literature

Since the rehabilitation phase with all three phases takes place over months if not years, multiple aspects are mentioned in the literature that might be challenging on the way to best possible recovery.

In the acute rehabilitation setting, the main challenges seem to be aspects such as delay in the rehabilitation chain including long-term linkage to rehabilitation practice. In addition, the multidisciplinary collaboration as well as the conduction of the acute rehabilitation in the hospital – potentially due to limited resources is considered a pitfall. Rehabilitation towards re-entry into work as well as further organizational/economical aspects play are demanding. Determining reliable assessment parameters for recovery also bears a challenge as well as individualized care and psychological impairments.

In the post-acute rehabilitation phase, especially the compliance and insight of the patient and their relatives plays a huge role for the proper conduction. Prior mentioned aspects such as maintaining the rehabilitation chain with focus on social and vocational rehabilitation still play an important role.

**Fig. 2:**
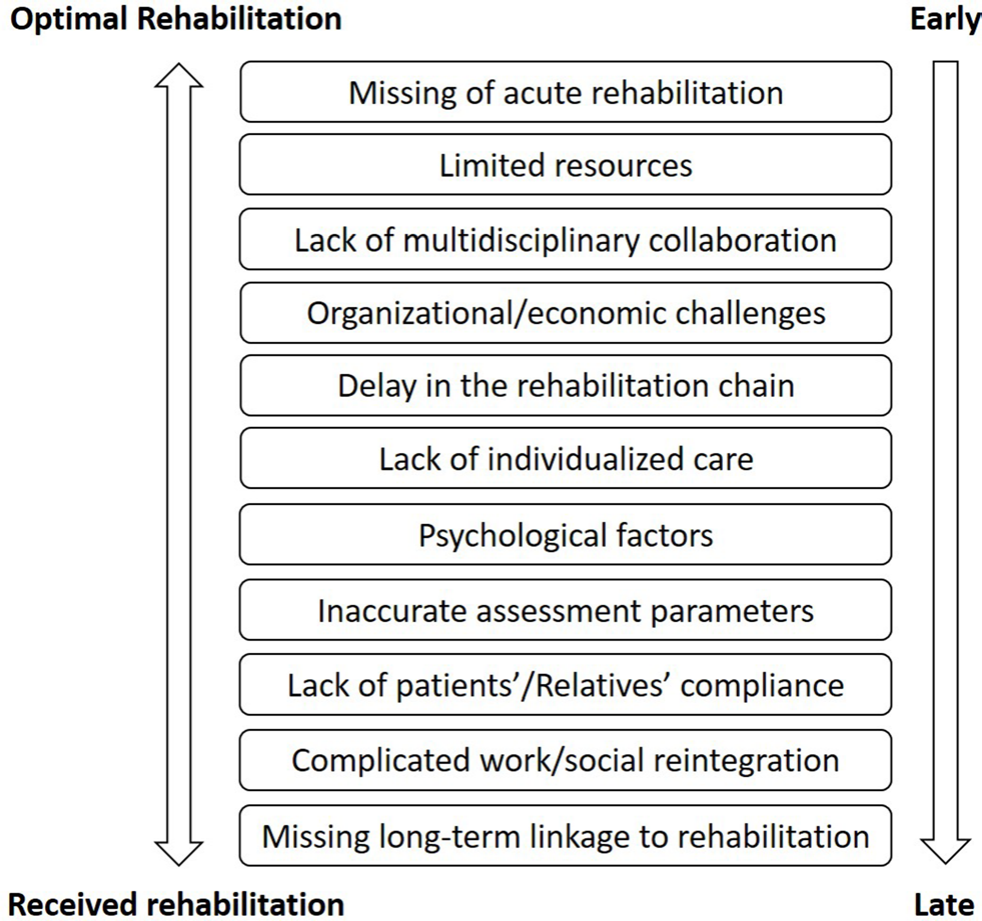
Pitfalls/challenges in the rehabilitation process

In the long-term rehabilitation, maintaining the proper rehabilitation chain remains demanding and requires proper compliance from the patient. Further, prior mentioned aspects remain important (Fig. 2; Table 4).

**Table 4:** Challenges during the rehabilitation stages

	Challenge	Acute stage stage					Post-acute stage		Long-term stage	
	Author/Publication	Debus et al.	Jang et al.	Matthey et al.	Simmel et al. 2010	Simmel et al. 2013	Critchfield et al.	Matthey et al.	Matthey et al.	Simmel et al. 2011
Acute rehabilitation conduction	Lack of acute rehabilitation in hospital	X		X	X					
Delay in rehabilitation chain	Delay of rehabilitation chain	X		X	X			X		
	Lack of rehabilitation management/coordination			X				X	X	
Long term linkage to rehabilitation	Need for long term care				X					
	Long term commitment (patients + providers)									X
	Addressing psychological and long-term consequences			X				X	X	
Multidisciplinary collaboration	Multidisciplinary collaboration challenges	X	X		X	X				X
Compliance/insight patients/relatives	Lack of insight in cognitive/physical limitations						X			
	Long term commitment (patients + providers)									X
	Patient motivation/compliance									X
	Potential tendency for substance abuse						X			
	Excessive relatives’ support restricts growth						X			
Limited resources	Resource constraints - professionals, facilities, equipment				X					X
Vocatioinal reintegration	Vocational rehabilitation, retraining, career changes			X	X			X	X	
Psychological	Psychological factors				X		X			X
Individualized care	Adequate individualized rehabilitation plan		X							X
Organizational/economic challenges	Economic, organizational and documentation challenges					X				
Inaccurate assessment	Inaccurate assessment		X			X	X			
Other	Staying up to date with rehabilitation advances									X

### Algorithm for polytrauma rehabilitation

**Fig. 3:**
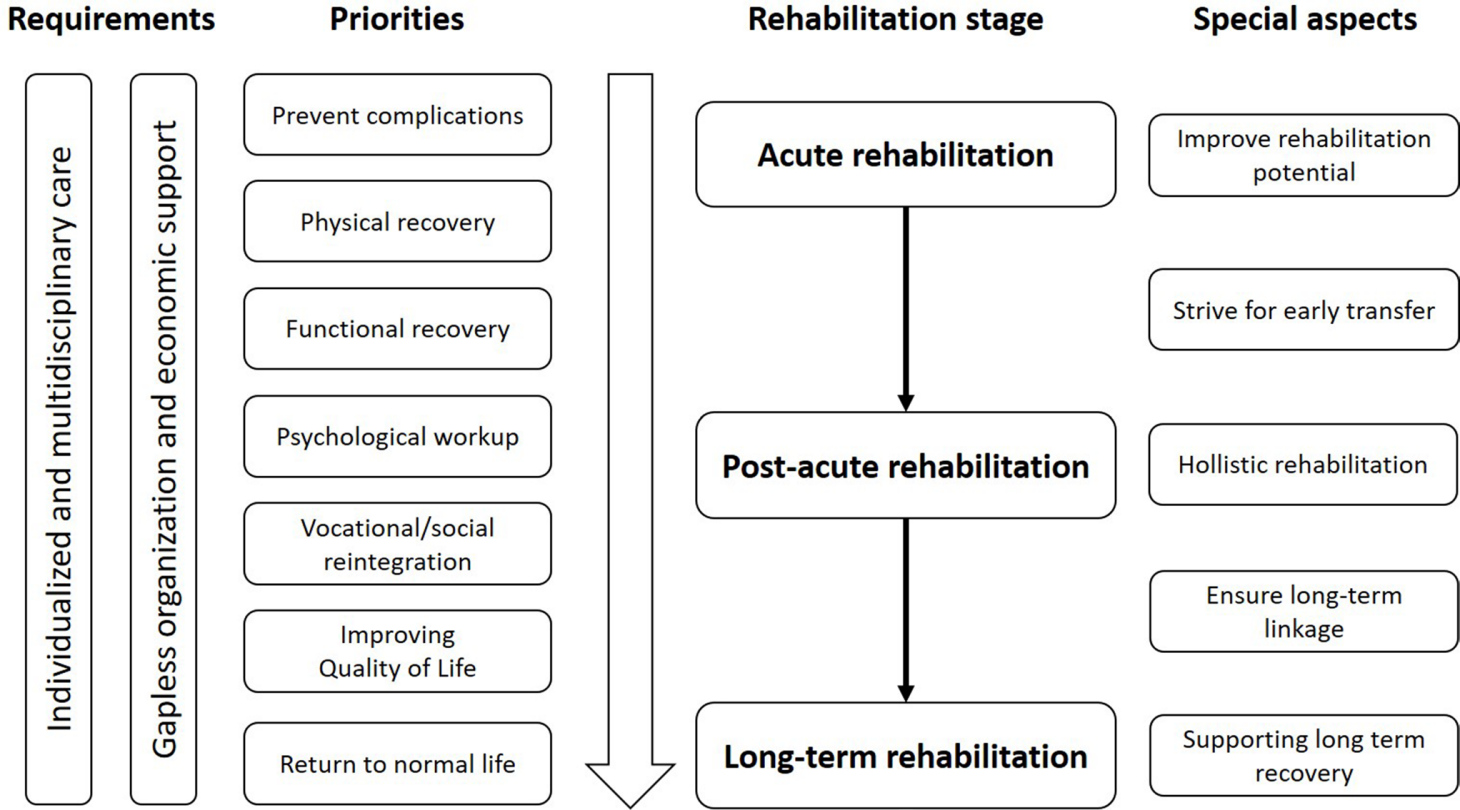
Proposed rehabilitation algorithm for polytraumatized patients

Based on the findings of this literature search, a comprehensive algorithm for the rehabilitation of polytraumatized patients was developed (Fig. 3).

## Discussion

Even though the rehabilitation plays a key part in the patient’s recovery after major trauma, this is mainly performed upon individual/regional clinic standards. Evidence-based literature on conduction of rehabilitation in polytrauma patients is rare to non-existent even though they are available for more isolated injury pattern. To a certain extent this is understandable, since standardized and reproducible research in this field is not easily possible, as polytrauma with all its varying definitions does not describe one specific injury pattern that can be targeted by one specific treatment approach but more a condition that is brought together by multiple injuries. Therefore, one would hardly be able to conduct any kind of randomized clinical trial on comparable patient cohorts with the aim to evaluate which one is more effective.

Yet, despite its obvious challenges, at least a standardized approach in the initial assessment and prioritization of different therapies is urgently required.

The reported literature in this article presents an overview of existing articles with emphasis on the different aspects stratified to the individual rehabilitation stage.

Based on the findings of this systematic review, we are able to formulate following main statement:

- There is great lack of clinical studies (as well as specific guidelines or recommendations) focusing on the rehabilitation in polytrauma patients.

The fact, that there is a lack of evidence in polytrauma rehabilitation has already been addressed by previous publications [3, 11]. Also in our extensive literature search, no clinical trial was identified that delivered elevated evidence on the rehabilitation in polytrauma patients. Sporadic literature was identified addressing the subjective feedback of patients and professionals on conducted polytrauma rehabilitation [12]. Yet, this is mainly focused on procedural procedures and self-perceptions that are difficult to objectify but provide important aspects to improve constantly the overall quality of rehabilitation.

Additionally, the authors could not identify specific recommendations in existing guidelines. Most guidelines on polytrauma care are focused on the initial phase and surgical/medical procedures or are focused on isolated injury patterns, which they provide specific rehabilitation advice on [13, 14]. One of the few more general recommendations identified were an immediate start of physio- and occupational therapy next to psychological support [15].

The “Rehabilitation gap/hole” is a term that is often referred to in the existing literature. It is meant as a loss of rehabilitation time on the road to recovery. This risk especially occurs in the interface between different rehabilitation stages as the patient for example does not present enough rehabilitation potential for post-acute rehabilitation or organizational hurdles occur to transfer the patient from acute rehabilitation [16]. The DGU^®^ proposed a new rehabilitation phase, called “early rehabilitation” that may bridge space between the acute and post-acute stage to strengthen the rehabilitation potential and might facilitate further therapy [17].

In our review of the literature, we were able to identify specific challenges that may occur during the rehabilitation journey and might impair the patient’s outcome. Those challenges should be tackled specifically by a multidisciplinary team, good organization and involvement of the patient in all belonging steps (Fig. 3).

In our review, we identified multiple therapies, aims and challenges in the respective rehabilitation phase. Following, we conclude the most important aspects in a description of an optimal rehabilitation process.

### Acute rehabilitation

Rehabilitation should start as early as possible respective to the patient`s injury pattern and interaction capability (intubation/cerebral deficits). Specialists of the involved clinics are required to give detailed restrictions and limitations, according to which the patient may be mobilized and trained. Based on this, the multidisciplinary rehabilitation team, preferably consisting of members of physiotherapy, occupational therapy etc. should conduct adjusted rehabilitation from the very beginning [4]. Depending on the patient’s status and impairments, further specialists for i.e. dysphagia/speech therapy and psychological workup can be involved [6, 7] (Table 3).

The initial aim in the acute phase is to (1) Improve the rehabilitation potential and (2) Prevent complications (i.e. thromboembolic events or pulmonary complications). If the patient cannot interact, passive movements of the extremities can be performed; otherwise, this can be done actively and depending on the injury pattern and related restrictions, mobilization in or outside the bed can be performed [18]. This should support DVT (deep vein thrombosis) preventions that should already exist respective to the injury pattern (i.e. intracranial bleeding) either medical (anticoagulation) or device supported (pneumatic cuffs) [19]. In addition, breathing/airway therapy should be conducted especially in the presence of thoracic injuries to prevent complications as pneumonia.

As soon as the required rehabilitation potential is reached and medical/surgical therapy is finished, the patient should be transferred to a specialized rehabilitation facility. This requires good interdisciplinary communication and professional management of the rehabilitation chain [4]. Delay of specialized rehabilitation due to organizational problems should be strongly avoided [8]. Also re-transfer back to the acute hospital due to complications is a major problem that occurs in around 8% of cases [20].

### Post-acute rehabilitation

The specialized rehabilitation has the potential to put the entire focus on the rehabilitation process, which is usually not possible in the acute hospital, where rehabilitation only takes place a short time a day. The aim in this “post-acute” setting is (1) Regaining physical function, (2) Setup for vocational/social reintegration and (3) Holistic therapy [8, 10]. Since the patient may not be able to pursue their former work even after completed rehabilitation due to disabilities or long-term functional limitations, social workers and vocational managers should be included into this process if not already done in the acute rehabilitation phase. Good involvement of the patient’s relatives is also important since they will play a major role especially after the patient is dismissed back into their former life [5]. Support in the social environment is required but may not exceed a certain level that the patient`s own growth is restricted. If psychological anomalies are observed or mentioned by the patient, a psychological consultancy should be conducted and followed up [8]. Consistent communication with initially treated hospital is essential to consult them in case, medical issues (i.e. infection, extraordinary pain, etc.) occur which might require a revision surgery or an intensive medical workup [20].

### Long-term rehabilitation

After the post-acute rehabilitation is finished and the patient is dismissed into their former life, a long-term rehabilitation is required. The aim hereby is to (A) Improve functionality to the best possible condition, (B) Support the patient in the vocational and social reintegration and (C) Assist in residual issues as (chronic) pain or dealing with disabilities [8]. Since this stage is usually performed in an outpatient physiotherapy setting, it requires good preparation to not lose track of the patient afterwards since it is the one that relies the most on the patient’s motivation [5]. Medical aftercare organized by the responsible department with regular visits of the consultation hours are also required to identify medical problems and to track with the rehabilitation process with potential further changes of initial restrictions. Also there might be a need for further surgeries according to the reconstruction plan and clinical/radiological follow up of the osseous healing process are required to identify potential problems.

## Conclusion

Rehabilitation after severe trauma represents a long journey to recovery with many obstacles of organizational or human genesis. An interdisciplinary team is required to plan and conduct all three stages of rehabilitation seamless with an individual therapy concept. More evidence on different rehabilitation strategies is urgently required in the field of polytraumatized patients.

## Electronic supplementary material

Below is the link to the electronic supplementary material.

Supplementary Material 1

## Data Availability

No datasets were generated or analysed during the current study.

## Abbreviations

DGU: Deutsche Gesellschaft für Unfallchirurgie
DVT: Deep vein thrombosis
PRISMA: Preferred Reporting Items for Systematic Reviews and Meta-Analyses
QOL: Quality of Life
SICOT: Société Internationale de Chirurgie Orthopédique et de Traumatologie
