# Supplementary material for: The three stages of polytrauma rehabilitation– a recommendation and a systematic literature review on behalf of SICOT
Source: Int Orthop. 2024 Dec 16;49(2):365–74. doi: 10.1007/s00264-024-06385-0 (PMC11762570; doi:10.1007/s00264-024-06385-0)
Supplement: Supplementary file 1 — Supplementary Material 1 [file 264_2024_6385_MOESM1_ESM.docx]

SI: Search Strategy of the systematic review

Databases: MEDLINE and Embase

Date of conducted search: 19.04.2023

**PEC(C)O Question**

| Concept | Physiotherapy in polytrauma patients |
| --- | --- |
| Patient, Population, Problem | Polytrauma patients |
| Exposure | Rehabilitation |
| Time Horizon | 2000-2023 |

**MEDLINE:**

"multiple trauma"[MeSH Terms] OR “polytrauma” [All Fields] OR “polytraumatized” [All Fields] OR “polytrauma*" [All Fields] OR “severe trauma” [All Fields] OR “severely injured"[All Fields] OR “multiple injured” [All Fields] OR “multiple injuries” [All Fields] OR “multiple trauma” [All Fields]

**AND**

"Physical Therapy Modalities"[Mesh] OR "Physical AND Rehabilitation Medicine"[Mesh] OR "Exercise"[Mesh] OR “Rehabilitation” [All Fields] OR “Aftercare” [All Fields] OR “Physiotherapy” [All Fields] OR “physiotherapies” [All Fields] OR “physical therapy” [All Fields] OR “physical therapies” [All Fields] OR “exercise therapy” [All Fields]

**AND**

(2000:2023[pdat])

**Results: 1517 publications**

**Embase:**

'multiple trauma'/exp OR 'polytrauma':ab,ti OR 'polytraumatized':ab,ti OR 'polytrauma*':ab,ti OR 'severe trauma':ab,ti OR 'severely injured':ab,ti OR 'multiple injured':ab,ti OR 'multiple injuries':ab,ti OR 'multiple trauma':ab,ti

AND

'rehabilitation center'/exp OR 'rehabilitation'/exp OR 'physical medicine'/exp OR 'aftercare'/exp OR 'physiotherap*':ab,ti OR 'physiotherapy':ab,ti OR 'rehabilitation':ab,ti OR 'physical therapy':ab,ti OR 'physical therapies':ab,ti OR 'exercise therapy':ab,ti

AND

[2000-2023]/py

**Results: 3675 publications**
